# Saturation Genome Editing Targeting *KRAS* Mutations in HCT 116 Colon Carcinoma Cells for Pooled SNV Functional Profiling in Diploid Cancer Model

**DOI:** 10.3390/cimb48040341

**Published:** 2026-03-25

**Authors:** Taiji Hamada, Seiya Yokoyama, Ryo Nakabayashi, Yoshihiko Suzuki, Shinichi Morishita, Toshiaki Akahane, Kei Matsuo, Ikumi Kitazono, Tatsuhiko Furukawa, Akihide Tanimoto

**Affiliations:** 1 Department of Pathology, Kagoshima University Graduate School of Medical and Dental Sciences, 8-35-1 Sakuragaoka, Kagoshima 890-8544, Japan; 2 Department of Computational Biology and Medical Sciences, Graduate School of Frontier Sciences, The University of Tokyo, 5-1-5 Kashiwanoha, Kashiwa 277-8563, Chiba, Japan; 3 Center for Human Genome and Gene Analysis, Kagoshima University Hospital, 8-35-1 Sakuragaoka, Kagoshima 890-8544, Japan; 4 Department of Surgical Pathology, Kagoshima University Hospital, 8-35-1 Sakuragaoka, Kagoshima 890-8544, Japan; 5 Center for Research of Advanced Diagnosis and Therapy of Cancer, Kagoshima University Graduate School of Medical and Dental Sciences, 8-35-1 Sakuragaoka, Kagoshima 890-8544, Japan

**Keywords:** saturation genome editing, variants of uncertain significance, *KRAS*, HCT 116 cells, colon carcinoma, *KRAS* ^G12C^ inhibitor

## Abstract

Evaluating cancer gene mutations is critical for effective therapeutic selection. Although massive parallel sequencing can efficiently detect gene mutations, most are variants of uncertain significance (VUS). Saturation genome editing (SGE) can facilitate VUS analysis by leveraging CRISPR-Cas9 and homology-directed repair to simultaneously introduce abundant gene mutations. Chronic myelogenous leukemia-derived HAP1 cells are widely used in SGE because of their clear genotype–phenotype relationship; however, the sole use of haploid cells limits SGE applicability in cancer research. Therefore, we developed an SGE-based system for evaluating *KRAS* mutations in diploid HCT 116 colon carcinoma cells. Single-nucleotide variants (SNVs) in *KRAS* codons A11–V14 were generated using Cas9-based SGE. Massive parallel sequencing revealed increased abundance of *KRAS* ^G12^ and *KRAS* ^G13^ SNVs and decreased abundance of the *KRAS* ^G12C^ SNV after *KRAS* ^G12C^ inhibitor treatment in SGE pooled cells. These results indicate that SGE is applicable to diploid HCT 116 cells and useful for evaluating SNV population changes and drug sensitivity. Thus, although haploid HAP1 cells are the primary models for SGE, the successful application of SGE to diploid HCT 116 colon carcinoma cells provides a practical framework for implementing SGE in *KRAS*-dependent carcinoma cells.

## 1. Introduction

Recent progress in massive parallel sequencing has improved the detection of genetic alterations in cancer. However, most gene alterations are single-nucleotide variants (SNVs) and variants of uncertain significance (VUS) [1,2,3]. Therefore, saturation genome editing (SGE), which simultaneously introduces multiple variants for genes of interest using the clustered regularly interspaced short palindromic repeats (CRISPR)-associated protein 9 (Cas9) system [4,5,6,7,8], can be a useful tool for VUS evaluation.

Most SGE studies are performed using chronic myelogenous leukemia-derived haploid HAP1 cells via homology-directed repair [4,5,6,7]. As genotypes clearly correspond to phenotypes in haploid cells, SGE in HAP1 cells helps elucidate the oncogenic significance of the variants. SGE-based mutational analysis relies on the principle that loss-of-function mutations in an essential gene lead to growth defects, and the gene of interest must be essential for cells [8]. Successful SGE depends on the nature of the cell and the selection of target genes. For example, SGE has been applied to malignant lymphoma-derived diploid TMD8 cells to investigate the function of *CARD11* in physiologically relevant contexts [9]. To the best of our knowledge, Cas9-based SGE via homology-directed repair using non-haploid carcinoma cells has rarely been reported. One exception is a study that introduced TP53 loss-of-function mutations in HCT 116 colon carcinoma cells [10]. Although SGE has recently been extended to diploid cell lines, most studies have primarily focused on loss-of-function assessments or tumor suppressor genes. In contrast, the functional interrogation of oncogenic gain-of-function variants remains insufficiently explored. HCT 116 cells harbor a heterozygous *KRAS* ^G13D^ mutation and exhibit *KRAS*-dependent proliferation. This provides a biologically relevant model in which variant fitness can be assessed under endogenous oncogenic signaling. Thus, this study was designed not merely to demonstrate technical feasibility in diploid cells but to evaluate whether SGE can quantitatively resolve mutation-specific proliferative advantages and drug sensitivities in HCT 116 cells.

In this study, we aimed to develop such a system by introducing common *KRAS* mutations [11]. As most *KRAS* mutations are gain-of-function mutations and significant drug targets, we selected diploid HCT 116 colon carcinoma cells, harboring *KRAS* ^G13D,^ as the dependent gene for proliferation [12]. Specifically, we introduced 36 SNVs in codons A11–V14 of *KRAS* exon 2. To evaluate *KRAS* functional alterations, edited HCT 116 cells were monitored for SNV populations and *KRAS* ^G12C^ inhibitor effects [13] to develop a potential system for SGE-based VUS assessment in non-HAP1 carcinoma cells.

## 2. Materials and Methods

### 2.1. Cell Culture

Human colon carcinoma HCT 116 cells were obtained from the RIKEN BioResource Research Center (Ibaraki, Japan). The cells were maintained in McCoy’s 5A medium (FUJIFILM Wako Pure Chemical, Osaka, Japan) supplemented with 2 mM glutamine, 100 U/mL penicillin, 100 μg/mL streptomycin, and 10% fetal bovine serum (Sigma-Aldrich, St Louis, MO, USA) and cultured at 37 °C with 95% air and 5% CO_2_.

### 2.2. Saturation Genome Editing

The cells were suspended in Opti-MEM (Thermo Fisher Scientific, Waltham, MA, USA) at a concentration of 1–2 × 10^7^ cells/mL. The Cas9 ribonucleoprotein complexes [3.2 μM, single-guide RNA (sgRNA): 5′-gtagttggagctggtgacgt; Integrated DNA Technologies (IDT), Coralville, IA, USA, Cas9; Nippon Gene, Tokyo, Japan] and single-strand oligodeoxynucleotide (3.4 μM, IDT) were added to 50 μL of cell suspension, then transferred into 2 mm cuvettes. Electroporation was conducted using a NEPA21 electroporator (Nepa Gene, Chiba, Japan) with 2× poring pulses (voltage 150 V; length 7.5 ms; interval 50 ms; polarity +) and 5× transfer pulses (voltage: 20 V; length: 50 ms; interval: 50 ms; polarity ±). Electroporated cells were seeded in six-well plates (Thermo Fisher Scientific) and subcultured every two or three days. At the indicated times, genomic DNA was extracted using a DNeasy Blood and Tissue Kit (QIAGEN, Hilden, Germany). For sotorasib (AMG510, Selleck, Houston, TX, USA) treatment, pooled cells five days after electroporation were incubated with 0.1 μM sotorasib and harvested 48 h after treatment.

### 2.3. Massive Parallel Sequencing

Genomic DNA was sequenced using a NextSeq 1000 sequencer (Illumina, San Diego, CA, USA) with a read coverage of 2–3 million reads per sample. The region of interest was amplified using PCR with a pair of forward (5′-TCTTTCCCTACACGACGCTCTTCCGATCTTTGTATTAAAAGGTACTGGTGGAGT-3′) and reverse (5′-GTGCAGGACCATTCTTTGATACAGAGATCGGAAGAGCACACGTCTGAACTCCAGTCAC-3′) primers. Genomic DNA (150 ng) was used as PCR template for each sample, corresponding to approximately 22,500 diploid cells (assuming 6.6 pg DNA per cell). A mixture of template DNA and the KOD One PCR Master Mix (TOYOBO, Osaka, Japan) was subjected to a PCR cycle (30 cycles at 98 °C for 10 s, 67 °C for 5 s, and 68 °C for 10 s). A second PCR was conducted with barcoding primer pairs, 1 μL of the first PCR product, and the KOD One PCR Master Mix (10 cycles of 98 °C for 10 s and 68 °C for 10 s). Equal molar amounts of secondary PCR products were mixed and purified using solid-phase paramagnetic beads (AMPure XP; Beckman Coulter, Brea, CA, USA).

### 2.4. Single Nucleotide Variant Analysis

SNV analyses were performed using R software (version 4.3.0). Packages for data handling (Biostrings, version 2.74.0) were used for sequence manipulation, and Tidyverse (version 2.0.0) provided dplyr for data wrangling and readr for efficient data import. Differential SNV abundance analysis was conducted using DESeq2 (version 1.40.2) [14] in a manner analogous to differential expression analysis, with visualization supported by ggplot2 (version 3.5.1), DEGreport (version 1.36.0), and EnhancedVolcano (version 1.18.0). Alignment of amplicon sequences to a reference sequence and quantification of editing efficiency were performed using CRISPResso2 (version 2.3.2) [15]. Raw sequence alignment data were searched for agreement with the donor sequence references, and the read counts of each SNV were extracted from the raw data. All reads corresponding to the target amplicon were included in the analysis without filtration of mutant and wild-type alleles. The calculated SNV frequencies represent combined variant abundance across both alleles and should be interpreted with caution when assessing allele-specific effects. For differential SNV abundance analysis, a DESeq DataSet object was constructed from the read counts and corresponding sample metadata, and SNV abundance was modeled as a function of time. Genes with a total read count below 10 across all samples were filtered to improve statistical power. Raw read counts were normalized using the median-of-ratios method implemented in DESeq2. Differential abundance was determined using the likelihood ratio test implemented in DESeq2 by comparing the full model (considering time points) against a reduced model (null hypothesis). Log_2_ fold changes (LFCs) were estimated using the negative binomial generalized linear model framework in DESeq2. For drug sensitivity analysis, normalized read counts were calculated using DESeq2, and normalized read counts after sotorasib treatment were compared with those without treatment. Statistical analysis of drug sensitivity was performed using R software. Data are presented as the means ± standard error. Statistical significance was determined using an unpaired two-tailed Student’s *t*-test, and the results were considered statistically significant at *p* < 0.05.

## 3. Results

### 3.1. Saturation Genome Editing of KRAS

**Figure 1:**
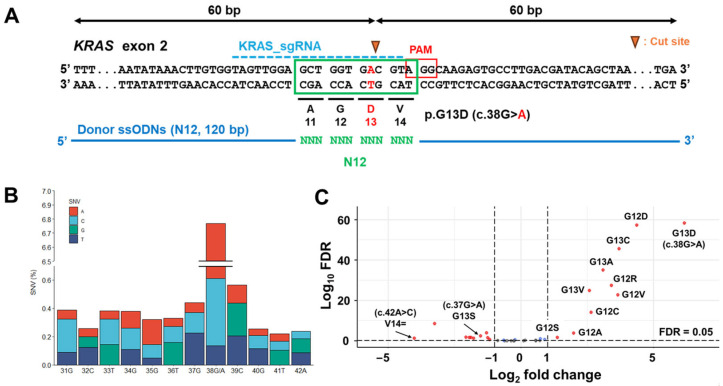
Saturation genome editing (SGE) of *KRAS* exon 2 in HCT 116 cells. (**A**) Schematic of the SGE strategy for *KRAS* codons A11 to V14 in HCT 116 cells, harboring *KRAS ^G13D^* (c.38G > A). Single-guide RNA (sgRNA) was designed at chr12: 25,245,344..25,245,363. For editing the allele with c.38G > A, the sixteenth guanine in sgRNA was replaced with adenine. Donor single-strand oligodeoxynucleotide (120 base pairs in length) comprised non-targeted strands containing the guide and protospacer adjacent motif (PAM) sequences. (**B**) Editing rates of the SGE region (31G to 42A base sequence corresponding to A11, G12, D13, and V14 of amino acids). Editing rates represent the mean percentage of total read counts in massive parallel sequencing analysis. (**C**) Volcano plot of differential mutation abundance in edited HCT 116 pooled cells. LFC values were estimated using DESeq2 based on a negative binomial generalized linear model with median-of-ratios normalization. Vertical dotted line represents LFC = 1 and −1. Horizontal dotted line represents the false discovery rate (FDR) = 0.05. Red points represent variants with |LFC| > 1 and FDR < 0.05, where FDR refers to the Benjamini-Hochberg-adjusted *p*-value as computed by DESeq2. All significance thresholds are FDR-based. SNV, single nucleotide variant; LFC, log_2_ fold change; FDR, false discovery rate; sgRNA, single-guide RNA; ssODN, single-strand oligodeoxynucleotides.

*KRAS* ^G12^ and *KRAS* ^G13^ variants are frequently detected in clinical sequencing and registered as oncogenic or likely oncogenic variants [16,17]; therefore, a target region was set at 12 bases from codons A11 to V14, generating a total of 36 SNVs (Figure 1A,B). As Cas9 ribonucleoprotein shows higher genome editing efficiency than Cas9-expressed plasmids in a glioblastoma cell line [18], we used the Cas9 ribonucleoprotein system for SGE in HCT 116 cells. The sgRNA was designed to preferentially target the mutant *KRAS* allele (*KRAS* ^G13D^, c.38G > A). Because the wild-type allele contains a mismatch within the sgRNA sequence, editing was expected to occur predominantly at the mutant allele. However, given the known tolerance of CRISPR–Cas9 to certain mismatches, editing of the wild-type allele cannot be completely excluded (namely, off-target effect). In the present study, the extent of off-target effects on the wild-type allele has not been quantitatively verified. We synthesized each single-strand oligodeoxynucleotide for SGE to 120 base pairs in length, extending on either side of the Cas9 cleavage site, and included a single SNV repair template and homology arms. To evaluate editing efficiency, genomic DNA was extracted three days after electroporation. All 36 SNVs were detected, and the editing rates ranged from 0.2 to 7.0% (Figure 1B); the editing rates of the two bases adjacent to the Cas9 cleavage site—38G/A and 39C—were higher than those of the other bases. As the original HCT 116 cells harbor a *KRAS* ^G13D^ mutation [11], c.38G > A coding *KRAS* ^G13D^ was the most frequent SNV.

### 3.2. KRAS ^G12/G13^ Mutations Enhanced Cell Growth

The abundance of SNVs across time points after SGE was quantified as the LFC using a likelihood ratio test. The LFC represents the magnitude and direction of change in SNV abundance. We performed SGE on quadruplet samples 13 days after electroporation. At 3, 5, 7, 10, and 13 days, genomic DNA was extracted from SGE pooled cells and subjected to massive parallel sequencing to calculate the LFC for each variant (Figure 1C). Higher LFC values reflect an expansion of selected SNVs under experimental growth conditions. Significantly higher LFCs were observed for *KRAS* ^G12^ and *KRAS* ^G13^ mutations, except for *KRAS* ^G13S^ (c.37G > A), indicating higher growth activities of these variants. Conversely, lower LFCs were observed for some *KRAS* ^A11^ and *KRAS* ^V14^ mutations, indicating that these variants did not affect cell growth under our experimental conditions. Synonymous substitutions (e.g., 33T > A and 33T > G) do not alter the encoded amino acid, but replace the pre-existing *KRAS* ^G13D^ (c.38G > A) allele. These variants thereby disrupt the oncogenic *KRAS* ^G13D^ context present in parental HCT 116 cells. Their depletion may thus be consistent with loss of *KRAS*-dependent proliferative signaling rather than a generalized deleterious effect of genome editing itself. All LFCs reflect pooled variant abundance across both alleles; the enrichment of *KRAS* ^G12^ and *KRAS* ^G13^ variants therefore represents selection at the cell-population level rather than allele-resolved effects.

### 3.3. Drug Sensitivity Test

**Figure 2:**
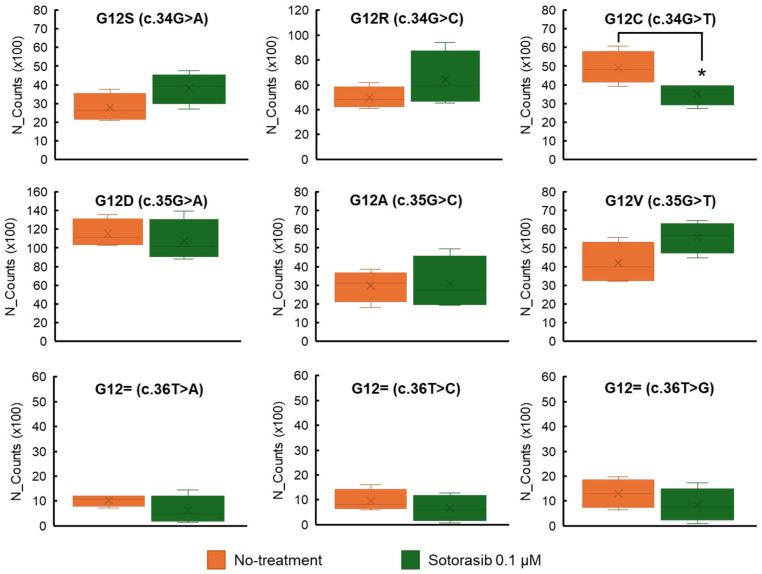
Normalized read counts of *KRAS* ^G12^ mutations. Edited pooled HCT 116 cells were treated with sotorasib (0.1 μM) for 48 h after five days of SGE. Normalized read counts of the *KRAS* ^G12C^ (c.34G > T) mutation in pooled cells were significantly decreased after sotorasib treatment. No significant changes were observed in the normalized read counts of other *KRAS* ^G12^ mutations. Normalized read counts were obtained using the median-of-ratios method implemented in DESeq2. N_Counts: normalized read counts; * *p* < 0.05 (unpaired two-tailed Student’s *t*-test).

SGE is suitable for *KRAS*-targeted drug testing because *KRAS* mutations are mostly single-base missense mutations [19]. Sensitivity tests for sotorasib (AMG510, a *KRAS* ^G12C^ covalent inhibitor) were performed using SGE-derived pooled cells. Normalized massive parallel sequencing read counts for each variant after sotorasib administration were compared to those without sotorasib. A significant decrease in normalized read counts was detected in *KRAS* ^G12C^ (c.34G > T), but not in the other *KRAS* SNVs (Figure 2). No effects of sotorasib were observed in *KRAS* ^G13^ variants, except *KRAS* ^G13V^, which showed increased read counts after sotorasib treatment (Figure S1). The precise reason for this increase in *KRAS* ^G13V^ abundance remains unclear; one possible explanation is that the GTP-binding affinity of the *KRAS* ^G13V^ mutant might be different from that of other *KRAS* ^G12/G13^ mutants.

## 4. Discussion

Most Cas9-based SGE studies via homology-directed repair are limited by the combination of haploid HAP1 cells and tumor suppressor gene loss-of-function mutations (Table 1) [4,5,6,7,9,10,20,21,22,23,24]. Although our SGE system introduced a smaller number of SNVs than previously reported SGE studies, we achieved successful SGE for *KRAS* gain-of-function mutations using diploid colon carcinoma HCT 116 cells. Importantly, the present study differs from previous diploid SGE applications in that the experimental system leverages an oncogene-addicted carcinoma background. This design enables the simultaneous evaluation of oncogenic gain-of-function variants and drug–variant interactions, thereby extending the functional utility of SGE beyond technical implementation in non-haploid cells.

In SGE applied to HCT 116 cells, most SNVs in *KRAS* ^G12^ and *KRAS* ^G13^ exhibited significantly higher LFC values, indicating an expansion of cells harboring specific SNVs. These findings are consistent with previous clinical sequencing data identifying *KRAS* ^G12^ and *KRAS* ^G13^ as hotspot mutations in human colorectal cancers [25,26]. Furthermore, SGE produced non-synonymous mutations, such as *KRAS* ^A11P^ and *KRAS* ^G12S^, which showed significantly lower LFC values. Under experimental growth conditions in HAP1 cells, *KRAS* is not essential for viability [27]. Therefore, SGE of *KRAS* in HAP1 cells would likely result in an unclear genotype–phenotype correlation and lower sensitivity for identifying pathogenic mutations. In contrast, *KRAS* mutation-dependent HCT 116 cells showed significant and discernible SGE outcomes. Kwon characterized *KRAS* variants using a deep mutational scanning (DMS) approach [28]. While DMS and SGE differ in whether mutations are assessed in an exogenous or endogenous context, the two approaches are complementary and will provide converging lines of evidence for the functional significance of *KRAS* mutations.

An important consideration of this study is the allelic context of genome editing in HCT 116 cells, which harbor a heterozygous *KRAS* ^G13D^ mutation. The sgRNA was designed to preferentially target the mutant allele; however, the editing of the wild-type allele cannot be entirely ruled out. Although we did not directly determine the allelic distribution of each editing event, the observed selection patterns are consistent with preferential modification of the mutant allele. Thus, while the genomic background is diploid, the functional readout may reflect editing dynamics that approximate a monoallelic-dominant context rather than fully balanced biallelic editing. Variant frequencies reflect pooled readouts across both alleles; accordingly, selection signals should be interpreted as operating at the level of the cell population rather than at the allele level.

As drug targets, gene mutations have significant impacts on drug efficacy [29]. In the post-genomic era, characterized by abundant VUS, analyzing individual gene mutations is inefficient. Therefore, comprehensive analyses of gene mutations and drug responses are fundamental to pharmacogenomic approaches. In this study, we demonstrated the efficiency of SGE in analyzing the effects of drugs on gene-mutated cells using edited pooled cells from a human epithelial cancer cell line. Because sotorasib exhibited an anti-proliferative effect only on cells harboring a target mutation among the 36 mutations, the SGE efficacy test has appropriate sensitivity for gene mutations. We expect that SGE-driven comprehensive high-throughput screening will improve the efficiency of molecularly targeted drug development and accelerate drug development. In our SGE model under sotorasib treatment, cells harboring *KRAS* ^G12C^ were selectively depleted, whereas other *KRAS* mutants were enriched. This pattern is consistent with clinical observations that loss of *KRAS* ^G12C^ and emergence of alternative RAS variants mediate adaptive resistance to sotorasib [30]. These findings support the utility of SGE-based drug sensitivity profiling for predicting clinically relevant resistance mechanisms.

A key extension of this approach is to apply the SGE-derived *KRAS* mutant pool to pan-RAS inhibitors. Unlike sotorasib, pan-RAS inhibitors target a broader spectrum of *KRAS* variants, making pooled SGE screening well-suited to identify resistance-conferring substitutions at G12 and G13. Such studies may reveal mutation-specific escape mechanisms relevant to clinical pan-RAS inhibition and inform combination strategies for *KRAS*-mutant cancers.

## 5. Conclusions

We demonstrate the feasibility of implementing an SGE system for colon carcinoma diploid HCT 116 cells, which exhibit a much more complex genomic background than haploid HAP1 cells and may reflect more physiological conditions.

## 6. Limitations

This study has several limitations. First, as HCT 116 cells are diploid, the editing outcomes for both alleles of each cell are unclear. Uncertain allelic editing outcomes may result in unclear associations between genotypes and phenotypes. The sgRNA is complementary to the mutant allele (c.38G > A) but not to the wildtype allele. However, given the known tolerance of CRISPR–Cas9 to certain mismatches, editing of the wild-type allele cannot be completely excluded. Therefore, the functional consequences observed in this study likely reflect editing that is enriched at the mutant allele, although the precise allelic distribution of edits was not directly resolved. In addition, as the protospacer adjacent motif (PAM) site in the sgRNA overlapped with the *KRAS* ^G13^ codon, no synonymous mutation was available within this overlapping region that could be introduced without altering the amino acid sequence or disrupting the PAM. This structural constraint makes it impossible to simultaneously introduce a synonymous mutation at the PAM site to prevent re-editing. The second limitation arises from the use of HCT 116 cells harboring a *KRAS* ^G13D^ (c.38G > A) heterozygous mutation. Although we focused on *KRAS* exon 2, an extension of SGE to other exons would require navigating this pre-existing mutation and a complicated experimental design, which may lower the genome editing efficiency to impractical levels. Third, the expansion of cells harboring specific SNVs and drug sensitivity was monitored by LFC, and a comparison of normalized SNV reads in edited pooled cells was made. Although this strategy is suitable for the first comprehensive evaluation of various SNV characteristics, a direct assessment of cloned cells is required to confirm the detailed characteristics of cancer cells.

## Figures and Tables

**Table 1:** Studies of Cas9-based SGE via HDR.

# Reference (Year)	GOI	Class of GOI	# SNVs	Delivery		Cell
				Cas9	Donors	
[4] (2014)	*BRCA1/DBR1*	TSG/-	234/225	plasmid	plasmid	HAP1
[5] (2018)	*BRCA1*	TSG	3893	plasmid	ssODN	HAP1
[9] (2020)	*CARD11*	OG	2542	RNP	plasmid	TMD8
[6] (2023)	*DDX3X*	TSG	12776	plasmid	plasmid	HAP1
[20] (2023)	*BRCA2*	TSG	851	plasmid	ssODN	mES
[21] (2024)	*RAD51C*	TSG	9188	HAP1-A5	plasmid	HAP1
[7] (2024)	*VHL*	TSG	2268	plasmid	plasmid	HAP1
[22] (2024)	*BAP1*	TSG	18108	HAP1-A5	plasmid	HAP1
[10] (2025)	*TP53*	TSG	9225	plasmid	plasmid	HCT 116*
[23] (2025)	*BRCA2*	TSG	6959	plasmid	plasmid	HAP1
[24] (2025)	*BRCA2*	TSG	6551	plasmid	plasmid	mES
present study	*KRAS*	OG	36	RNP	ssODN	HCT 116

#, number; Cas9, clustered regularly interspaced short palindromic repeats (CRISPR)/CRISPR-associated protein 9; SGE, saturation genome editing; HDR, homology-directed repair; GOI, gene of interest; TSG, tumor suppressor gene; OG, oncogene; mES, mouse embryonal stem cell; RNP, ribonucleoprotein; ssODN, single-strand oligodeoxynucleotide. HCT 116* cells were silenced in one TP53 allele.

## Data Availability

The datasets generated during the current study are available through the DNA Data Bank of Japan (DDBJ) database (https://www.ddbj.nig.ac.jp (accessed on 24 February 2026)) under accession number PRJDB37492.

## Supplementary Materials

The following supporting information can be downloaded at: https://www.mdpi.com/article/10.3390/cimb48040341/s1.

## Abbreviations

The following abbreviations are used in this manuscript:

Cas9	Clustered regularly interspaced short palindromic repeats (CRISPR)-associated protein 9
FDR	False discovery rate
GOI	Gene of interest
HDR	Homology-directed repair
LFC	Log_2_ fold change
mES	Mouse embryonal stem cell
OG	Oncogene
PAM	Protospacer adjacent motif
PCR	Polymerase chain reaction
RNP	Ribonucleoprotein
SGE	Saturation genome editing
sgRNA	Single-guide RNA
SNV	Single-nucleotide variant
ssODN	Single-strand oligodeoxynucleotide
TSG	Tumor suppressor gene
VUS	Variants of uncertain significance
