# Supplementary material for: Saturation Genome Editing Targeting KRAS Mutations in HCT 116 Colon Carcinoma Cells for Pooled SNV Functional Profiling in Diploid Cancer Model
Source: Curr Issues Mol Biol. 2026 Mar 25;48(4):341. doi: 10.3390/cimb48040341 (PMC13115350; doi:10.3390/cimb48040341)
Supplement: Supplementary file 1 [file cimb-48-00341-s001.zip › cimb-4196909-supplementary.pdf]

Supplementary Materials

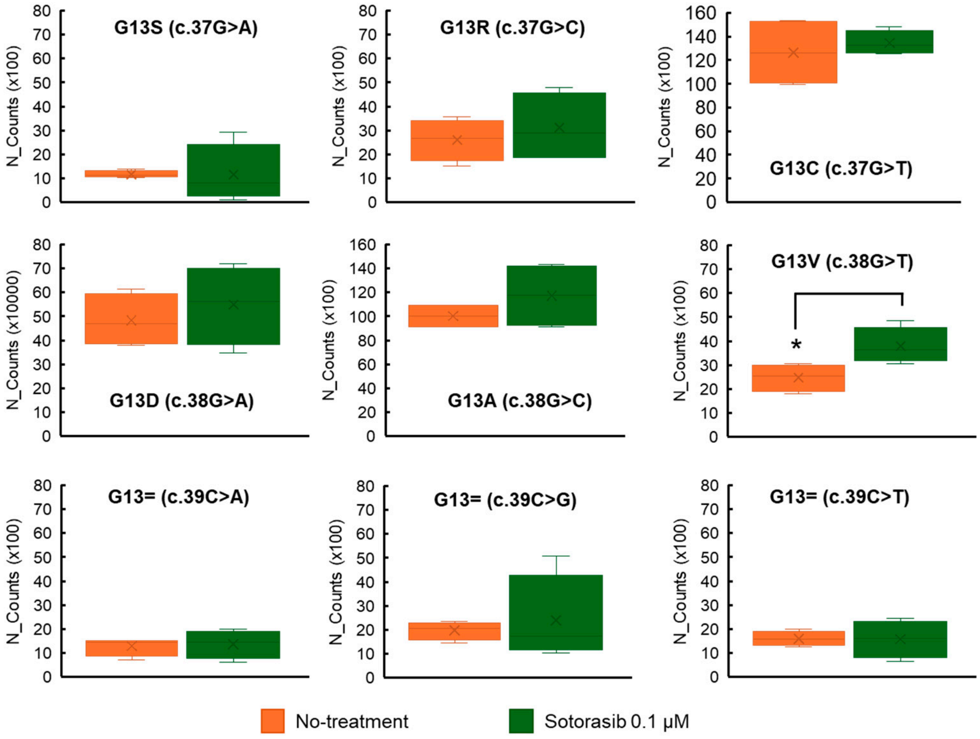

Figure S1. Normalized read counts of *KRAS* ^{G13} mutations. No significant changes were observed in the normalized read counts of *KRAS* ^{G13} mutations in pooled cells with or without sotorasib treatment, except for *KRAS* ^{G13V}. The precise reason for this increase in *KRAS* ^{G13V} abundance remains unclear; one possible explanation is that a GTP-binding affinity of the *KRAS* ^{G13V} mutant might be different from that of other *KRAS* $^{G12/G13}$ mutants. N_Counts: normalized read counts; * $p < 0.05$ (unpaired two-tailed Student's t-test).
